# The riddle of lymphoma BCR-antigenes

**DOI:** 10.18632/oncotarget.26318

**Published:** 2018-11-09

**Authors:** Lorenz Thurner, Sylvia Hartmann, Klaus-Dieter Preuss, Moritz Bewarder

**Affiliations:** José Carreras Center for Immuno- and Gene Therapy and Internal Medicine I, Saarland University Medical School, Homburg, Germany

**Keywords:** lymphoma-B cell receptor, chronic BCR activation, mantle cell lymphoma, primary central nervous system lympphoma, B-cell receptor antigen for reverse targeting [BAR]

The hypothesis of antigen-triggered chronic B cell receptor (BCR) stimulation in lymphoproliferative disorders is old and has gained further popularity in recent years [1], [2]. Currently, we reported in two articles on the frequent reactivity of BCRs of mantle cell lymphoma to LRPAP1 and BCRs of primary central nervous system lymphoma to SAMD14 / neurabin-I [3], [4]. We have demonstrated that LRPAP1, a protein that is expressed in almost all human tissues, is a common target antigen of B cell receptors of mantle cell lymphomas. 8 of 21 primary MCL cases expressed a BCR that showed reactivity against LRPAP1. Among the MCL cases with BCR-reactivity against LRPAP1, both subtypes with mutated and unmutated variable regions of immunoglobulin (IGV) genes were found. It was also shown that two of seven established MCL cell lines have an LRPAP1-reactive BCR. Whether the BCR reactivity against LRPAP1 has an influence on the prognosis is not yet clear. Of interest, patients with LRPAP1-reactive MCL-BCRs had serum-autoantibodies against LRPAP1, which is suggestive of a chronic autoimmune response. The reason for the loss of immune tolerance in these MCL cases against LRPAP1 is unclear. In contrast to several targets of paraproteins possessing atypical posttranslational modifications such as sumoylation or hyperphosphorylation [5], [6], no such secondary changes have been found in LRPAP1.

For PCNSLs we were able to identify SAMD14 and the highly homologous SAM domain of neurabin-I as the autoantigenic targets of the BCR of approximately 67% of all cases. Here, in contrast to LRPAP1, specific posttranslational modifications of the BCR-target antigens were identified - N-hyperglycosylation of SAMD14 at N339 and of neurabin-I at N1277. N-hyperglycosylated SAMD14 and neurabin-I as determined from PCNSL cryosections were exclusively found in patients with lymphoma BCR-reactivity against SAMD14 / neurabin-I. However, neither the PCNSL-BCRs nor SAMD14/neurabin-I autoantibodies present in these patients were glyco-specific, i.e. BCRs and autoantibodies bound to hyperglycosylated SAMD14 / neurabin-I as well as to wild type SAMD14 / neurabin-I.

Several questions arise with these intriguing findings:

First, it is an area of speculation why LRPAP1 is the specific BCR-antigen of MCL and SAMD14/neurabin-I of PCNSL and why HSP90 and SLP2 are cognate antigens for plasma cell dyscrasia [7], [8]. Chronic auto-antigenic stimulation may explain to some extent lymphoma genesis from a polyclonal immune response against described proteins, but the reasons for the loss of self-tolerance remain elusive. Posttranslational modification seems a reasonable explanation for this process but can not account for the immune response to LRPAP1 and its role in MCL pathogenesis.

Closely related to the first question is the question whether subtypes of other lymphoma entities might bear similar specific BCR-reactivities. Chronic-BCR stimulation by auto- and infectious antigens in MALT lymphoma, stereotyped specific target antigens in CLL, and autoreactive BCRs in ABC-type DLBCL have been demonstrated or suspected [9], [10], [11], [12], [2]. In context with this, new discoveries of lymphoma BCR antigen targets for DLBCLs, subforms of Burkitt's lymphoma and Hodgkin's lymphoma are expected. It is unclear whether EBV-positive lymphoma, which are often considered to be BCR-independent due to LMP2a [13], also rely in part on direct BCR-antigen interaction and whether patients with immunosuppression-associated CNS lymphoma could also show BCR-reactivity to SAMD14/neurabin-I.

The second important question is whether the detection of elevated titers of serum-antibodies to LRPAP1 or SAMD14/neurabin-I could predict the development of an MCL or PCNSL, and if so, with what predictive power and at which time interval. The relative risks, that we observed in our studies, indicate at least high specificity of these autoantibodies but further studies with larger case numbers are necessary to determine the prognostic impact of such antibodies. If confirmed, preemptive measures such as B cell depletion in patients with elevated autoantibody titers might be discussed.

A third question is whether the BCR-reactivity of lymphoma is just another piece of the puzzle in the pathogenesis of certain subgroups of lymphoma or if this biased BCR-reactivity could be used as a therapeutic target.

For this, it will be crucial to know whether lymphoma use resistance mechanisms simply via their AID mutation machinery. If this were not the case, the concept of eradication of a certain B-cell population with a specific BCR-reactivity by BCR-antigen/immunotoxins could be transferred from autoimmunity to lymphoma research [14]. Here, surface Ig of lymphoma cells bind the cognate epitope, which is fused to a toxin. After binding this B-cell receptor antigen for reverse targeting (BAR), the immunotoxin is internalized into the lymphoma cell and the toxic payload is thus specifically released within.

This concept is similar to the concept of BCR-targeting by anti-idiotype antibodies. However, an advantage over anti-idiotype antibodies would be the possibility to use the same constructs inter-individually in different patients. Furthermore, in addition to BCR-epitope/toxin fusion proteins described in current studies, more progressive concepts such as BITEs or CAR-T cells with the lymphoma-BCR-epitopes as baits, i.e. as ectodomain of CARs would offer new therapeutic approaches. It has been previously shown in mouse models of pemphigus vulgaris with CAR-T cells with desmoglein 3 ectodomains in the presence of desmoglein-3 autoantibodies, that these concepts could function even in the presence of autoantibodies [15].

In the case of lymphoma-BCRs with specificity to the posttranslationally modified epitope, such as sumoylated HSP90, the generation of BAR incorporating therapeutics might be more challenging. However, lymphoma BCRs were not specific for posttranslationally modified isoforms in the majority of lymphoma-BCR antigens identified so far [4], [3], [7].

The described lymphoma BCR-antigens were identified by modified SEREX-approaches established by Michael Pfreundschuh and his group. The BAR concept was passionately supported by him.

We very much regret that he cannot continue his work together with us on these projects. His enduring support, intellectual brilliance as well as he himself as a human being will be missed.
